# Prediction of moderate-to-severe postoperative thirst in general anesthesia patients based on automated machine learning and clinical nursing translation

**DOI:** 10.3389/fmed.2026.1846230

**Published:** 2026-09-14

**Authors:** Yi Zhan, Siting Yang, Haoran Wang

**Affiliations:** Department of Anesthesiology and Perioperative Medicine, The First Affiliated Hospital with Nanjing Medical University, Nanjing, Jiangsu, China

**Keywords:** automated machine learning, enhanced recovery after surgery (ERAS), explainable artificial intelligence, general anesthesia, postoperative thirst, risk prediction

## Abstract

**Objective:**

To develop an Automated Machine Learning (AutoML)-based model for screening Moderate-to-Severe Postoperative Thirst (MSPOT) risk at surgery-to-PACU handover and a prototype clinical decision support system.

**Methods:**

This retrospective single-center cohort included 928 patients undergoing general anesthesia (training set: *n* = 650; held-out internal test set: *n* = 278). An Improved Wave Optics Optimizer (IWOO) framework integrated feature selection and hyperparameter optimization using demographic, preoperative, and intraoperative variables. Prediction was performed at anesthesia-to-PACU handover, after final intraoperative data became available and before routine postoperative NRS thirst assessment. Performance was evaluated using ROC-AUC, PR-AUC, calibration analysis, Brier score, and decision curve analysis (DCA). SHAP was used to summarize feature contributions.

**Results:**

In the held-out internal test set, the AutoML model achieved a ROC-AUC of 0.9053, PR-AUC of 0.9080, and Brier score of 0.129, outperforming conventional models. DCA showed greater net benefit across thresholds of 1%–95%. In same-center temporal validation, discrimination remained acceptable (ROC-AUC: 0.8807; PR-AUC: 0.8778), but calibration deteriorated (Brier score: 0.1924; intercept: −1.2250; slope: 0.5811), indicating average risk overestimation and overly extreme probabilities. Six predictors were identified: esmolol use, intraoperative blood loss, operation time, ASA classification, ERAS, and intraoperative fluid volume.

**Conclusions:**

The AutoML model showed strong discrimination and transparent feature attribution. The prototype illustrates its potential use at PACU handover to prioritize early assessment and comfort-oriented thirst management. However, temporal miscalibration indicates that individualized probabilities are not ready for clinical decision-making without recalibration and subsequent prospective validation.

## 1 Introduction

Moderate-to-Severe Postoperative Thirst (MSPOT) is a common and distressing symptom in patients recovering from general anesthesia and can adversely affect the immediate postoperative care experience (1). Perioperative thirst has been described as an intense, often underrecognized symptom accompanied by dry mouth, dry lips, thick saliva, dry throat, unpleasant taste, and a strong desire to drink (2, 3). These patient-centered burdens underscore the importance of timely assessment and safe symptom management in perioperative care (4). Current postoperative thirst management practices primarily rely on passive interventions guided by nursing experience (such as lip moistening with cotton swabs or minimal water intake), lacking timely identification immediately after surgery and precise prevention pathways for high-risk patients (5). Therefore, constructing a high-precision, interpretable, and clinically translatable MSPOT prediction model is urgently needed to achieve postoperative risk stratification at PACU admission and guide targeted post-anesthesia care, thereby advancing the new methodology of perioperative comfort care.

Previous studies on MSPOT predominantly focused on exploring single risk factors within traditional epidemiological frameworks. While these studies revealed associations between thirst and factors such as age, operation duration, anesthetic agents, and fluid balance (6–9), they suffer from three major limitations (10–14): First, traditional statistical methods (e.g., logistic regression, chi-square tests) inadequately address complex nonlinear interactions among multiple variables, particularly demonstrating weak feature extraction capability for high-dimensional clinical data. Second, conventional risk prediction models (e.g., nomograms) heavily depend on researcher-prespecified variable inclusion sequences and statistical inference assumptions, easily introducing subjective bias and reducing model robustness. Most notably, such models face significant clinical implementation barriers: on one hand, model parameters lack transparent explanatory mechanisms to clarify the intrinsic logic of feature contributions; on the other hand, existing prediction tools based on classical machine learning (e.g., SVM, random forests) require physicians to possess strong programming skills for data inference and result interpretation, hindering their integration into routine clinical workflows and ultimately leading to a disconnect between research value and practical application. These shortcomings highlight the urgent need for innovative technical methodologies in the MSPOT prediction field to bridge the gap between model development and real-world implementation.

Machine Learning (ML) technology offers a novel methodological pathway to address these bottlenecks, with its core advantage lying in the algorithm's autonomous ability to learn deep data patterns. In prognostic prediction, ML models excel by automatically capturing nonlinear correlations and higher-order interactions through feature engineering. This capability often results in superior data modeling and prediction accuracy compared to traditional models (15, 16). As a subfield, Automated Machine Learning (AutoML) further utilizes meta-heuristic algorithms to achieve synergistic automatic optimization of feature selection and hyperparameter tuning, effectively reducing technical barriers to modeling while mitigating performance volatility risks inherent in manual parameter adjustment. More importantly, model interpretability techniques represented by SHAP (Shapley Additive Explanations) can transform algorithmic “black-box” decisions into quantifiable explanations of prediction outcomes, visualizing feature contributions and directional impacts multidimensionally to provide clinicians with reliable theoretical support for risk-informed decision-making (17–19). These technical characteristics grant ML tremendous potential in developing intelligent tools that integrate high predictive performance with clinical acceptability.

Building upon this foundation, this study aims to systematically address key challenges in predicting and optimizing intervention strategies for MSPOT in general anesthesia patients through the integration of innovative ML frameworks and clinical workflow design. We will: (1) Develop a precision prediction model based on an improved AutoML algorithm to enable risk stratification during the immediate postoperative transition to PACU and validate its performance superiority over traditional models; (2) Introduce interpretable modeling techniques to analyze core clinical feature clusters associated with MSPOT occurrence and improve the transparency of model prediction logic; (3) Construct a visual decision support system designed for potential integration into PACU handover and early assessment workflows, aiming to enhance healthcare quality and patient satisfaction.

## 2 Methods

### 2.1 Study population and data collection

This retrospective cohort study consecutively enrolled adult patients undergoing elective general anesthesia surgery at The First Affiliated Hospital with Nanjing Medical University from January 2023 to March 2024, who were subsequently transferred to the Post-Anesthesia Care Unit (PACU). All data were extracted from the hospital's Electronic Medical Record (EMR) system. Ethical approval was obtained (Approval No.: 2024-05035), with informed consent waived due to the retrospective nature. No formal prospective study protocol was prepared or registered before study initiation.

Inclusion criteria: Age ≥ 18 years, elective general anesthesia surgery, PACU transfer, and complete perioperative data. Exclusion criteria: Emergency surgery, local/regional anesthesia, direct ICU transfer, severe cognitive impairment, or incomplete clinical data/key variable deficiencies. Among 928 ultimately enrolled patients, a 7:3 ratio was applied to randomly allocate them into training (*n* = 650) and test (*n* = 278) sets.

Postoperative sedation status was assessed using the Riker Sedation-Agitation Scale, while thirst intensity was measured via the Numeric Rating Scale (NRS, 0–10 scale; ≥4 defined MSPOT). Variables collected encompassed: (1) Demographics: Age, sex, education level; (2) Preoperative factors: ERAS implementation, daily water intake, preoperative fasting duration, preoperative bowel preparation; (3) Surgical/anesthetic factors: Operation/anesthesia duration, surgical approach, ASA classification, intubation method; (4) Intraoperative fluid management: Infusion volume, crystalloid/colloid ratio, blood loss, urine output; (5) Anesthetic agents: 12 thirst-related agents (e.g., Esmolol, Propofol) filtered from 79 drugs (Prior to model development, we performed a pre-specified, outcome-independent screening of 79 perioperative medications documented in the electronic medical records. The screening criteria encompassed pharmacological and clinical relevance, traceability of drug records, exposure frequency, and non-zero variance of the exposure variables; duplicate drug names and different salt forms were also standardized. This screening process did not use the moderate-to-severe postoperative thirst outcome, univariate *P* values, outcome association effect sizes, or any information obtained after the outcome had occurred. Ultimately, 12 medication variables with clinical plausibility and available exposure information were retained. The specific screening rules and drug list are provided in Supplementary Table S7 of Appendix A. All subsequent feature selection that involved outcome information was confined to resampling or cross-validation procedures within the training set; the held-out internal test set and temporal validation cohort were not involved in this process.); (6) Outcome: MSPOT. To evaluate the model's robustness and generalizability over time, we additionally established a same-center temporal validation cohort. This cohort consecutively enrolled 152 adult patients who underwent elective general anesthesia surgery and were subsequently transferred to the PACU at the same medical center between April 2024 and August 2024. Its inclusion and exclusion criteria, perioperative data collection methods, and postoperative thirst intensity assessment methods remained completely consistent with those of the model development cohort.

Data collection included electronic medical record extraction and standardized bedside assessment of postoperative thirst intensity within 30 min after PACU admission. Thirst intensity was assessed by a trained research nurse using the Numerical Rating Scale (NRS, 0–10, where 0 represents no thirst and 10 represents the worst imaginable thirst). The primary outcome, moderate-to-severe postoperative thirst, was defined as an NRS score ≥ 4. For routine care, direct NRS assessment remains the reference approach when patients are sufficiently awake and able to communicate. The proposed model is intended to provide an early risk flag during PACU admission and handover when immediate patient-reported assessment may be delayed by residual sedation, airway devices, nausea, or workflow constraints. Because complete outcome assessment was required for model development, patients were included only when a reliable NRS score could be obtained within the prespecified 30 min window after PACU admission. All assessors received standardized training on the assessment protocol prior to study initiation, and periodic consistency checks were conducted to ensure inter-rater reliability. No predictor variable exhibited completely random missingness; upon verification, the individual missing proportion for each variable ranged from 0.2% to 4.3%, all below the pre-specified threshold of 5%. Among these, the variables with relatively higher missing rates were preoperative daily water intake (4.3%) and intraoperative urine output (3.8%), while the missing rates for the remaining variables were all below 2.5%. Given the limited missing proportions and the absence of any variable with ≥5% missingness, this study did not initiate multiple imputation procedures; instead, a single imputation strategy appropriate for low-proportion missingness was adopted: continuous variables were imputed with the median, and categorical variables were imputed with the mode. After imputation, the distribution characteristics (mean, standard deviation, and frequency proportions) of each variable showed no significant deviation compared with the complete observations before imputation, suggesting that this simple imputation method did not introduce discernible systematic bias.

Prior to estimating any preprocessing parameters, the 928-case development cohort was first stratified by outcome and randomly divided into a training set (*n* = 650) and a held-out internal test set (*n* = 278); the 152-case temporal validation cohort was separately retained by enrollment time and did not participate in model development. For each internal cross-validation fold, the median of continuous variables and the mode of categorical variables were estimated solely from the training portion of that fold, and the same estimates were applied unchanged to the validation portion of that fold. After model development was completed, the final preprocessing parameters were re-estimated using the full training set and applied unchanged to the held-out internal test set and temporal validation cohort. The category levels of categorical variables, one-hot encoding rules, and the order of encoded columns were likewise determined and fixed solely from the training data. The test set and temporal validation cohort did not participate in imputation value estimation, encoding rule determination, feature selection, hyperparameter tuning, threshold determination, or model coefficient estimation. The specific data isolation and preprocessing workflow is detailed in Supplementary Table S3 of Appendix A, and the one-hot encoding mapping is provided in Supplementary Table S6 of Appendix A.

### 2.2 Automated machine learning model development

We constructed an automated machine learning framework based on the Improved Wave Optics Optimizer (IWOO), aiming to achieve the synchronous selection of the optimal feature subset and the fine-tuning of core hyperparameters in a data-driven manner. The original WOO algorithm performs global exploration by simulating the phenomenon of light diffraction and local exploitation by simulating the phenomenon of interference, while IWOO is enhanced in the following two key aspects. In the population initialization phase, we adopted a Logistic chaotic mapping instead of purely random initialization to ensure that the initial candidate solutions possess traversal uniformity and dispersion diversity within the search space. In the local exploitation phase, when searching according to the interference principle, the algorithm embeds a Lévy flight strategy: leveraging its long-tail distribution characteristics, it periodically executes large-step jumps during the process of fine searching with a majority of small steps, thereby effectively breaking out of local optimum traps and enhancing the global search capability. The effectiveness of this improved algorithm was validated using the CEC2022 benchmark function set.

The entire AutoML pipeline process is as follows. First, the dataset containing 28 candidate variables was included, and categorical variables were one-hot encoded. IWOO performed a two-phase synchronous optimization: the first was discrete feature space screening, modeling feature selection as a binary optimization problem, where each candidate solution contained a 0/1 encoded binary vector, with 0 indicating exclusion and 1 indicating inclusion; the second was continuous hyperparameter space optimization, which, for the base learner XGBoost, automatically tuned its six key hyperparameters: n_estimators [range (50, 500)], max_depth [(3, 15)], learning_rate [(0.01, 0.3)], subsample [(0.6, 1.0)], colsample_bytree [(0.6, 1.0)], and gamma [(0, 0.5)]. The optimized objective function was to maximize the ROC-AUC under five-fold cross-validation within the training set. The termination condition was set as reaching the maximum number of iterations (200) or the improvement of the population's optimal fitness being less than a tolerance of 1 × 10^−6^ for 15 consecutive generations. After optimization, the final AutoML model was trained on the full training data using the output optimal feature subset and hyperparameters. To clearly separate each phase, we emphasize that all the above feature selection and hyperparameter tuning only utilized the training set data, with the test set being completely isolated during this process. Subsequently, all comparison models—Logistic Regression (LR), Support Vector Machine (SVM), AdaBoost, XGBoost, and LightGBM—also obtained their optimal configurations and completed training on the training set through a grid search hyperparameter optimization method (The exact candidate hyperparameter grid and their values are provided in Supplementary Table S5 of Appendix A). They were then evaluated together with the AutoML model on the held-out internal test set, which was never involved in any development process, and finally underwent same-center temporal validation, yielding the final reported metrics. This environment was developed in MATLAB 2024b. The pseudo-code for the AutoML model is seen in Appendix A. The technical roadmap of the study is seen in Figure 1. To ensure the stability and reproducibility of feature selection, all swarm intelligence algorithms (IWOO, WOO, WOA, GWO, PSO, GA, GA-PSO, and GA-ACO) were independently run 100 times on the training set. For each algorithm, if a candidate feature appeared in the final feature subset in more than 50% of the 100 independent runs (i.e., selection probability > 0.5), it was deemed selected by that algorithm. The feature selection results of all algorithms are displayed as a selection-frequency matrix in Figure 2.

**Figure 1:**
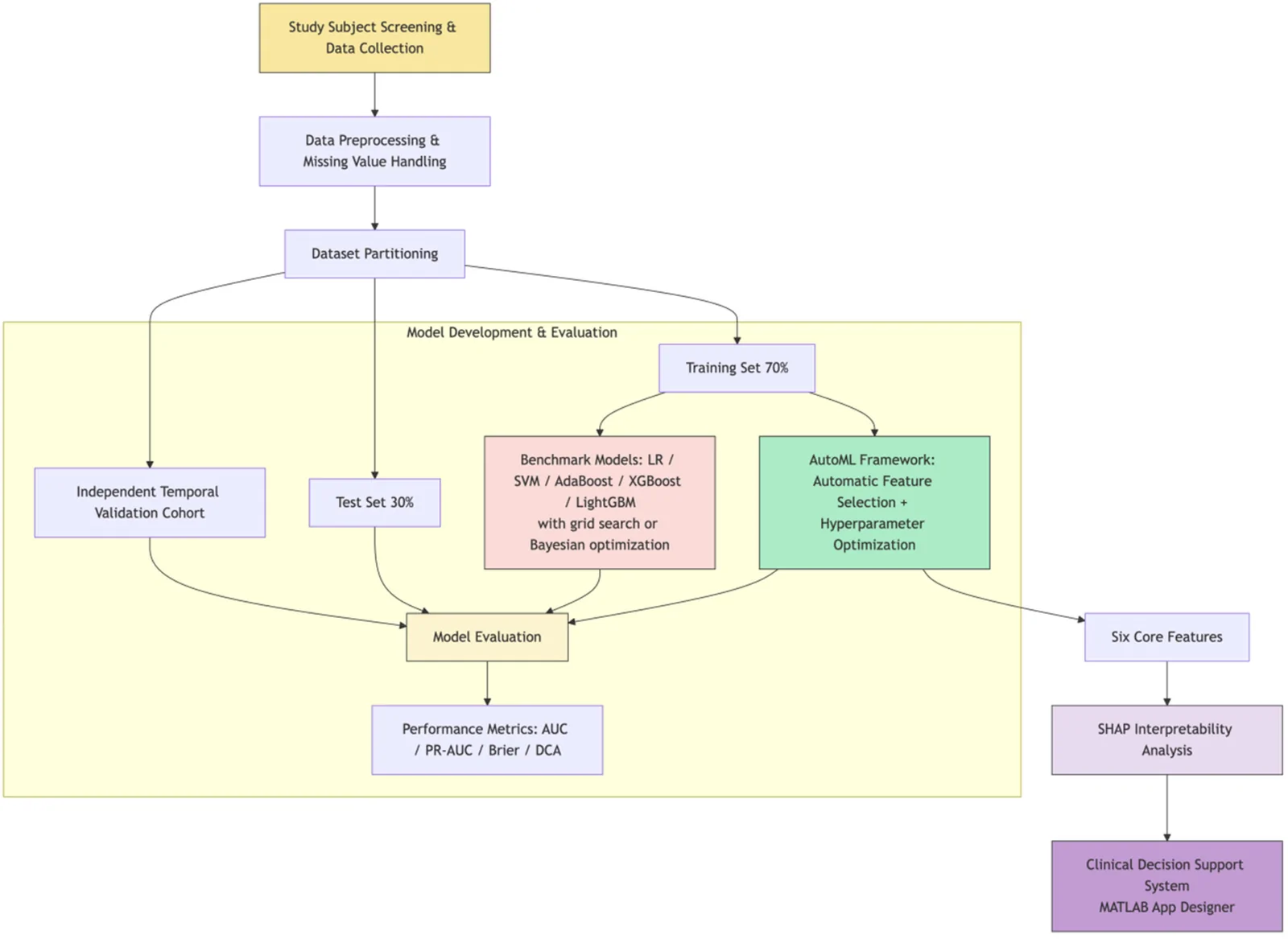
Technical workflow diagram.

**Figure 2:**
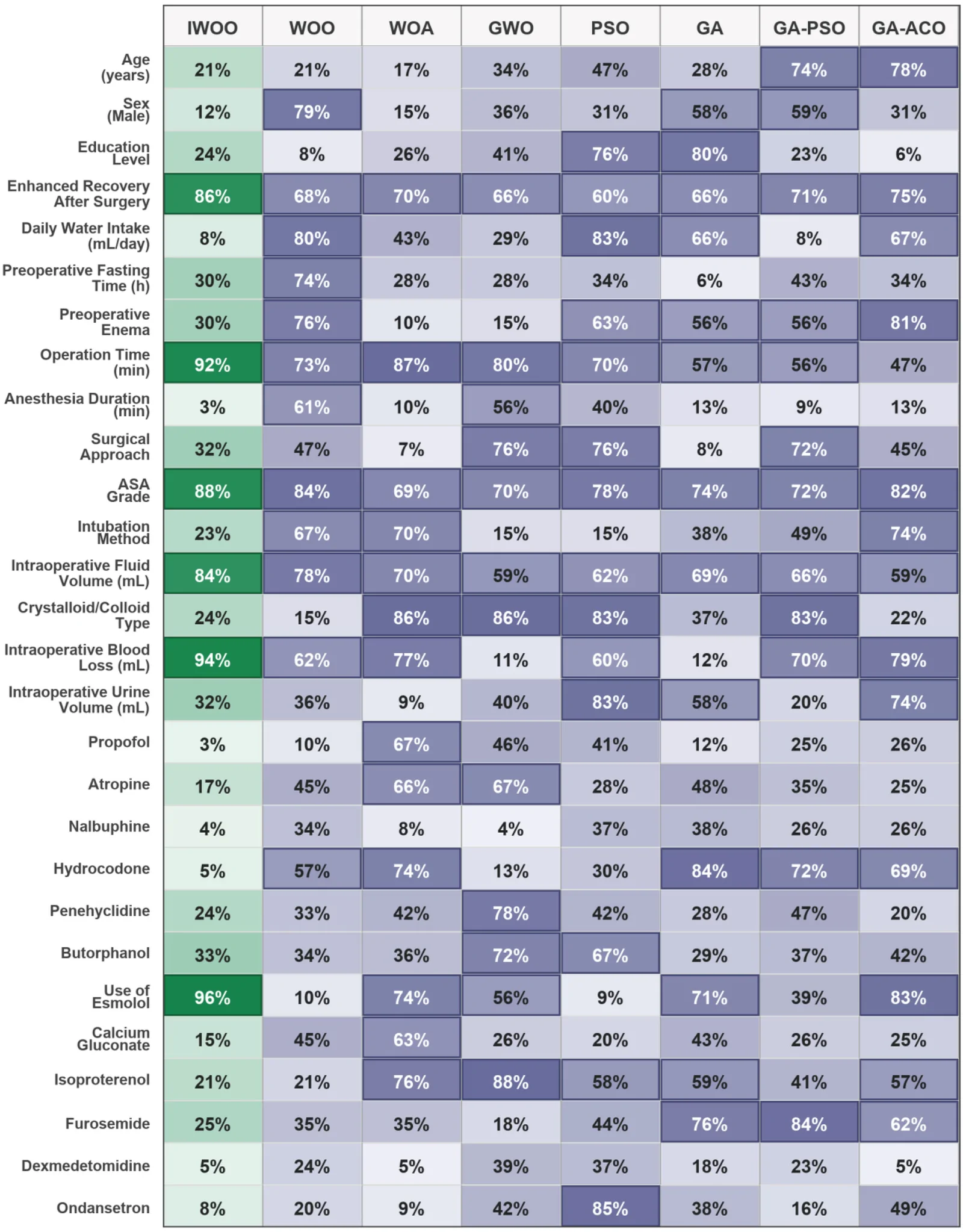
Feature selection frequency across swarm intelligence algorithms. This study evaluated the feature selection performance of the IWOO algorithm and seven other swarm intelligence optimization algorithms (WOO, WOA, GWO, PSO, GA, GA-PSO, GA-ACO). Each cell shows the actual selection frequency (%) of the corresponding feature across 100 independent runs on the training set. Frequencies > 50% were predefined as stable selection and correspond to features retained by each algorithm. Darker colors indicate higher selection frequency; thick borders indicate frequencies > 50%. The number of retained variables was treated as an indicator of model compactness rather than, by itself, proof of better feature-selection performance.

To enhance the reproducibility of the analysis, the IWOO optimization process adopted fixed optimization settings: a population size of 30, a maximum of 200 iterations, and early termination when the change in the swarm's best fitness was less than 1 × 10^−6^ for 15 consecutive generations. The optimization objective was the mean ROC-AUC under stratified five-fold cross-validation within the training set. The random seeds used for data splitting, cross-validation, IWOO optimization, and bootstrap confidence interval calculation are respectively recorded in Supplementary Table S3 of Appendix A. The final XGBoost hyperparameters and their search ranges are provided in Supplementary Table S4 of Appendix A, and the one-hot encoding mapping for the 28 candidate variables and the finally retained feature blocks are provided in Supplementary Table S6 of Appendix A. The AutoML pseudocode is given in Appendix A, and the complete results and test settings of the CEC2022 benchmark are shown in Supplementary Tables S1,S2 of Appendix B.

### 2.3 Evaluation metrics

The evaluation of all models strictly adhered to the principle of separating development and validation. Test set data did not participate in any feature selection, hyperparameter specification, or coefficient estimation processes; they were only used in the final stage to calculate the following metrics, thereby ensuring an unbiased estimate of generalizability. Each model outputs a prediction probability between 0 and 1, which reflects the individual's risk of developing moderate-to-severe postoperative thirst. To convert the continuous probability value into a binary classification decision, this study uniformly adopted 0.5 as the classification threshold: when the predicted probability ≥ 0.5, it was classified as positive; when the predicted probability < 0.5, it was classified as negative. In the proposed PACU workflow, this high-risk flag would prompt prioritization of direct NRS reassessment once reliable communication is possible, intensified oral comfort care, and safety-appropriate thirst-relief measures according to local postoperative swallowing and aspiration-risk protocols. A multidimensional evaluation framework was established: (1) Classification performance: Assessed using six core metrics for prognostic prediction models: Accuracy (ACC), Sensitivity (SEN), Specificity (SPE), F1-score (harmonic mean of precision and recall), Area Under the Receiver Operating Characteristic Curve (ROC-AUC), and Area Under the Precision-Recall Curve (PR-AUC). These metrics systematically evaluated model discrimination ability and stability under class imbalance; (2) Calibration performance: Evaluated using calibration curves and the Brier score (lower values indicate higher prediction accuracy). Calibration curves were generated by grouping predicted probabilities into ten approximately equal-sized bins and plotting the observed MSPOT incidence against the mean predicted probability within each bin; (3) Clinical utility: Clinical utility was quantified using Decision Curve Analysis (DCA), calculating the Net Benefit (NB) across a clinically relevant range of threshold probabilities. Based on the clinical context of PACU thirst management—where interventions are generally low-risk and a low threshold for action may be acceptable—the threshold probability range for DCA was restricted to 1% to 95%. The NB is defined as:

NB=(TP/N)−(FP/N)*[p_t/(1−p_t)]

where TP denotes true positives, FP denotes false positives, N denotes the total sample size, and p_t denotes the risk-threshold probability. The model's effective clinical decision-making range was validated by comparing NB against traditional intervention strategy reference lines. Within this range, the model's effective clinical decision-making value was assessed by comparing NB against the “treat all” and “treat none” reference strategies.

### 2.4 Interpretability analysis

After initial feature selection via the AutoML framework, LASSO regression was applied to validate the robustness of the selected features. Subsequently, the SHapley Additive exPlanations (SHAP) model was used to analyze feature clinical plausibility. The specific workflow comprised: (1) AutoML feature pre-screening: Utilizing predefined search spaces and optimization objectives, the AutoML algorithm automatically identified the feature subset most significantly associated with prognosis; (2) LASSO feature validation: LASSO regression was applied to the AutoML-selected subset. Its sparsity and stability were verified through the regularization constraint mechanism, ensuring feature resistance to overfitting. Differences between LASSO-selected and AutoML-selected features were compared; (3) SHAP interpretability analysis: By calculating Shapley values for each feature, quantitative attribution of feature contributions to individual predictions was achieved; summary plots were used to display global feature importance rankings, while waterfall plots and a decision path plot were retained to illustrate representative prediction patterns. These SHAP visualizations were interpreted as explanations of model behavior rather than evidence of causal effects.

### 2.5 Interactive tool development

To demonstrate proof-of-concept for future clinical translation, the App Designer module in MATLAB 2024b was used to develop a prototype Clinical Decision Support software tool. The prototype was designed for use at PACU admission after the final intraoperative variables have been entered or imported from the anesthesia record. Given the miscalibration observed in the temporal validation cohort, the current interface should be interpreted as a research prototype for displaying risk ranking and workflow concepts; its individualized probability outputs require recalibration and prospective evaluation in a separate dataset before clinical use.

### 2.6 Statistical analysis

Data were uniformly imported into the SPSS 26.0 statistical platform. Continuous variables conforming to a normal distribution are presented as mean ± standard deviation (x¯ ± s), while non-normally distributed continuous variables are presented as median with interquartile range [M (Q1, Q3)]. Categorical variables are presented as frequency and percentage (*n* %). For inter-group comparisons: Continuous variables underwent normality testing; if both groups conformed to normality, an independent samples t-test was used; otherwise, the Mann–Whitney U test was applied. Categorical variable comparisons employed Pearson's chi-square test. Statistical significance was determined using *P*-values (two-tailed test, significance threshold *α* = 0.05). Results are structured in tables for presentation.

## 3 Results

### 3.1 Participant characteristics

Baseline characteristics of the training and test sets are summarized descriptively in Table 1. Because the test set was generated by random splitting from the same development cohort, these baseline summaries were used only to describe the distribution of variables across subsets and were not interpreted as evidence of model generalizability.

**Table 1:** Baseline characteristics of participants.

Characteristic	Training set (*n* = 650)	Test set (*n* = 278)
MSPOT, *n* (%)		
Yes	363 (55.85%)	147 (52.88%)
No	287 (44.15%)	131 (47.12%)
Demographic		
Age (years), M [Q1, Q3]	52 [42, 63]	53 [41, 62]
Male, *n* (%)		
Yes	336 (51.69%)	149 (53.60%)
No	314 (48.31%)	129 (46.40%)
Education level, *n* (%)		
Primary school or below	97 (14.92%)	37 (13.31%)
Junior high school	168 (25.85%)	74 (26.62%)
Senior high school	220 (33.85%)	94 (33.81%)
College degree or above	165 (25.38%)	73 (26.26%)
Preoperative factors		
ERAS, *n* (%)		
Yes	299 (46.00%)	125 (44.96%)
No	351 (54.00%)	153 (55.04%)
Daily water intake (mL/day), mean ± SD	1,791.28 ± 396.30	1,746.11 ± 379.92
Preoperative fasting time (hours), M [Q1, Q3]	8.4 [6.7, 10.1]	8.5 [6.9, 10.2]
Preoperative bowel preparation, *n* (%)		
Yes	240 (36.92%)	100 (35.97%)
No	410 (63.08%)	178 (64.03%)
Surgical and anesthetic information		
Operation time (minutes), M [Q1, Q3]	96.5 [70.2, 122.0]	91.8 [61.5, 118.7]
Anesthesia duration (minutes), M [Q1, Q3]	115.2 [88.2, 148.8]	124.7 [89.6, 147.6]
Surgical approach, *n* (%)		
Laparoscopic	251 (38.62%)	108 (38.85%)
Open	216 (33.23%)	88 (31.65%)
Endoscopic	103 (15.85%)	52 (18.71%)
Other	80 (12.31%)	30 (10.79%)
ASA classification, *n* (%)		
ASA I	95 (14.62%)	41 (14.75%)
ASA II	311 (47.85%)	133 (47.84%)
ASA III	206 (31.69%)	91 (32.73%)
ASA IV	38 (5.85%)	13 (4.68%)
Intubation method, *n* (%)		
Endotracheal intubation	485 (74.62%)	211 (75.90%)
Laryngeal mask	134 (20.62%)	44 (15.83%)
Other	31 (4.77%)	23 (8.27%)
Intraoperative fluid management		
Intraoperative fluid infusion (mL), M [Q1, Q3]	1,550 [1,143, 1,970]	1,547 [1,100, 1,859]
Crystalloid/colloid infusion type, *n* (%)		
Crystalloid only	301 (46.31%)	137 (49.28%)
Colloid only	59 (9.08%)	34 (12.23%)
Both	282 (43.38%)	100 (35.97%)
None	8 (1.23%)	7 (2.52%)
Intraoperative blood loss (mL), M [Q1, Q3]	120 [60, 200]	120 [65, 210]
Intraoperative urine output (mL), M [Q1, Q3]	396 [254, 518]	420 [266, 538]
Anesthetic drugs		
Propofol, *n* (%)		
Yes	545 (83.85%)	225 (80.94%)
No	105 (16.15%)	53 (19.06%)
Atropine, *n* (%)		
Yes	177 (27.23%)	65 (23.38%)
No	473 (72.77%)	213 (76.62%)
Nalbuphine hydrochloride, *n* (%)		
Yes	198 (30.46%)	88 (31.65%)
No	452 (69.54%)	190 (68.35%)
Hydrocodone hydrochloride, *n* (%)		
Yes	108 (16.62%)	35 (12.59%)
No	542 (83.38%)	243 (87.41%)
Penehyclidine hydrochloride, *n* (%)		
Yes	141 (21.69%)	64 (23.02%)
No	509 (78.31%)	214 (76.98%)
Butorphanol tartrate, *n* (%)		
Yes	158 (24.31%)	68 (24.46%)
No	492 (75.69%)	210 (75.54%)
Esmolol, *n* (%)		
Yes	220 (33.85%)	92 (33.09%)
No	430 (66.15%)	186 (66.91%)
Calcium gluconate, *n* (%)		
Yes	125 (19.23%)	49 (17.63%)
No	525 (80.77%)	229 (82.37%)
Isoprenaline, *n* (%)		
Yes	73 (11.23%)	27 (9.71%)
No	577 (88.77%)	251 (90.29%)
Furosemide, *n* (%)		
Yes	96 (14.77%)	41 (14.75%)
No	554 (85.23%)	237 (85.25%)
Dexmedetomidine hydrochloride, *n* (%)		
Yes	263 (40.46%)	120 (43.17%)
No	387 (59.54%)	158 (56.83%)
Ondansetron hydrochloride, *n* (%)		
Yes	323 (49.69%)	146 (52.52%)
No	327 (50.31%)	132 (47.48%)

### 3.2 Performance evaluation of improved swarm intelligence algorithm

After evaluation with standard test functions, the IWOO algorithm demonstrated clear superiority in global optimization capability (Appendix B). In the real clinical data, all swarm intelligence algorithms were independently run 100 times on the training set. Figure 2 reports the actual feature-level selection frequency for each algorithm across these 100 runs; features with selection frequency > 50% were retained by the corresponding algorithm. As shown in the feature selection-frequency matrix, IWOO retained six predictive features with selection frequencies ranging from 84% to 96%. Comparator algorithms retained 12.7 features on average, indicating that IWOO produced a more compact feature subset; however, compactness alone was not interpreted as evidence of superior feature selection. The selected subset was therefore considered together with cross-validation performance, temporal validation, LASSO consistency analysis, and SHAP-based clinical plausibility.

### 3.3 Model training outcomes

Six machine learning models were systematically evaluated on the training set (Table 2). The AutoML model demonstrated superior performance with ROC-AUC: 0.9315 and PR-AUC: 0.9357 (Figures 3A,B). Notably, its F1-score (0.8919) indicates stronger clinical utility in precision-recall balance. AutoML ultimately identified six key features: Use of Esmolol, Intraoperative blood loss, Operation time, ASA classification, ERAS, and intraoperative fluid infusion volume. The optimal hyperparameters of the final AutoML model are presented in Supplementary Table S4 in Appendix A.

**Table 2:** Performance evaluation metrics of prediction models.

Dataset	Model	PRE	SEN	SPE	ACC	F1	ROC-AUC (95%*CI*)	PR-AUC (95%*CI*)	DeLong test (Z/P) compared with AutoML
Training set	LR	0.7271	0.8953	0.5749	0.7538	0.8025	0.8100 (0.7722–0.8444)	0.8179 (0.7783–0.8525)	6.143/<0.001
	SVM	0.7336	0.8953	0.5889	0.7600	0.8065	0.8039 (0.7641–0.8402)	0.8120 (0.7708–0.8493)	6.521/<0.001
	AdaBoost	0.8072	0.8650	0.7387	0.8092	0.8351	0.8636 (0.8332–0.8907)	0.8776 (0.8480–0.9041)	4.102/<0.001
	XGBoost	0.8391	0.8623	0.7909	0.8308	0.8505	0.8942 (0.8681–0.9173)	0.8997 (0.8740–0.9228)	2.875/0.004
	LightGBM	0.8396	0.8650	0.7909	0.8323	0.8521	0.9062 (0.8829–0.9271)	0.9130 (0.8901–0.9334)	2.237/0.025
	AutoML	0.8653	0.9201	0.8188	0.8754	0.8919	0.9315 (0.9102–0.9504)	0.9357 (0.9121–0.9551)	-
Test set	LR	0.6404	0.8844	0.4427	0.6763	0.7429	0.7505 (0.6941–0.8034)	0.7635 (0.7022–0.8183)	5.429/<0.001
	SVM	0.6298	0.8912	0.4122	0.6655	0.7380	0.7272 (0.6703–0.7809)	0.7213 (0.6584–0.7794)	5.817/<0.001
	AdaBoost	0.8042	0.7823	0.7863	0.7842	0.7931	0.8236 (0.7728–0.8691)	0.8320 (0.7772–0.8779)	3.204/0.001
	XGBoost	0.7081	0.8912	0.5878	0.7482	0.7892	0.8454 (0.7995–0.8856)	0.8550 (0.8061–0.8967)	2.489/0.013
	LightGBM	0.6816	0.9320	0.5115	0.7338	0.7874	0.8292 (0.7786–0.8735)	0.8292 (0.7747–0.8749)	2.781/0.005
	AutoML	0.8355	0.8639	0.8092	0.8381	0.8495	0.9053 (0.8691–0.9383)	0.9080 (0.8684–0.9420)	—

Confidence intervals were calculated based on 2,000 bootstrap resamples; the DeLong test was used to compare the ROC-AUC differences between each respective model and the AutoML model.

**Figure 3:**
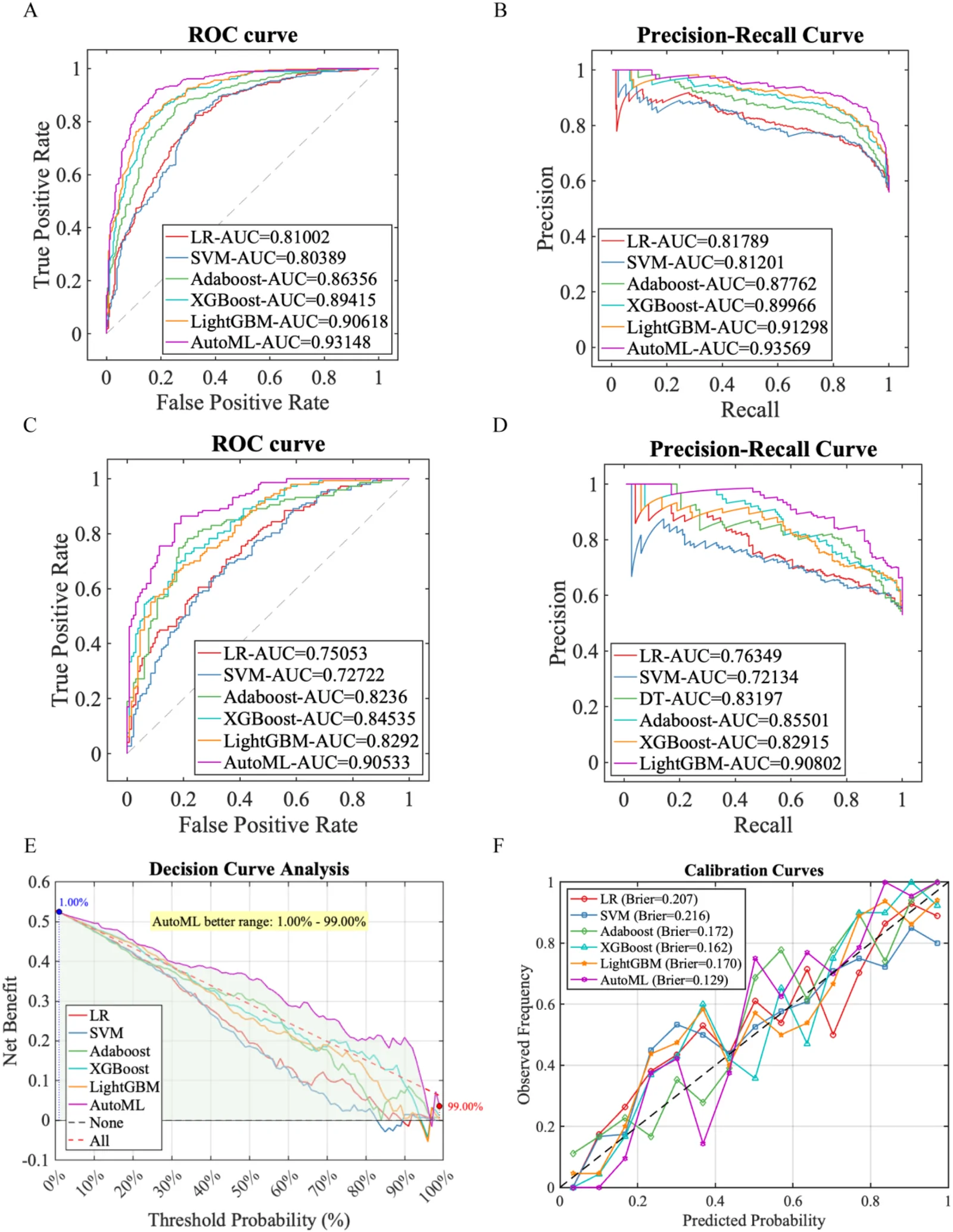
Model training performance and validation. **(A)** ROC curve (training set); **(B)** PR curve (training set); **(C)** ROC curve (test set); **(D)** PR curve (test set); **(E)** DCA curve (test set); **(F)** Calibration curve (test set). Calibration curves were constructed using decile-based observed event rates; bootstrap 95% confidence intervals for calibration metrics are reported in the Results.

During held-out internal testing, AutoML maintained robust performance, achieving a ROC-AUC of 0.9053 and a PR-AUC of 0.9080 (Figures 3C,D). Decision curve analysis (Figure 3E) demonstrated that AutoML provided greater net clinical benefit compared to traditional methods across the 1%–95% risk threshold range. Calibration analysis (Figure 3F) confirmed its superior predictive accuracy, yielding the lowest Brier score of 0.129. To more comprehensively evaluate the uncertainty surrounding key performance metrics, we further applied 2,000 bootstrap resamples to calculate the 95% confidence intervals for all threshold-dependent performance and calibration metrics of the AutoML model in the held-out internal test set. The model achieved a sensitivity of 0.8639 (95% CI: 0.7982–0.9151), specificity of 0.8092 (95% CI: 0.7380–0.8684), accuracy of 0.8381 (95% CI: 0.7894–0.8812), and an F1-score of 0.8495 (95% CI: 0.8018–0.8917). In terms of calibration, the 95% confidence interval for the Brier score ranged from 0.0987 to 0.1623, the calibration slope had a 95% confidence interval of 0.8815–1.2236 (point estimate of 1.0520), and the calibration intercept had a 95% confidence interval spanning −0.1482 to 0.0861 (point estimate of −0.0312). Furthermore, in the decision curve analysis, taking a risk threshold probability of 20% as an example, the net benefit had a point estimate of 0.4519, with a 95% confidence interval of 0.3821–0.5224. These interval estimation results further corroborated the robustness of the AutoML model across discrimination, calibration, and clinical utility dimensions. The confusion matrices of the prediction results for each model on the test set are shown in Figure 4.

**Figure 4:**
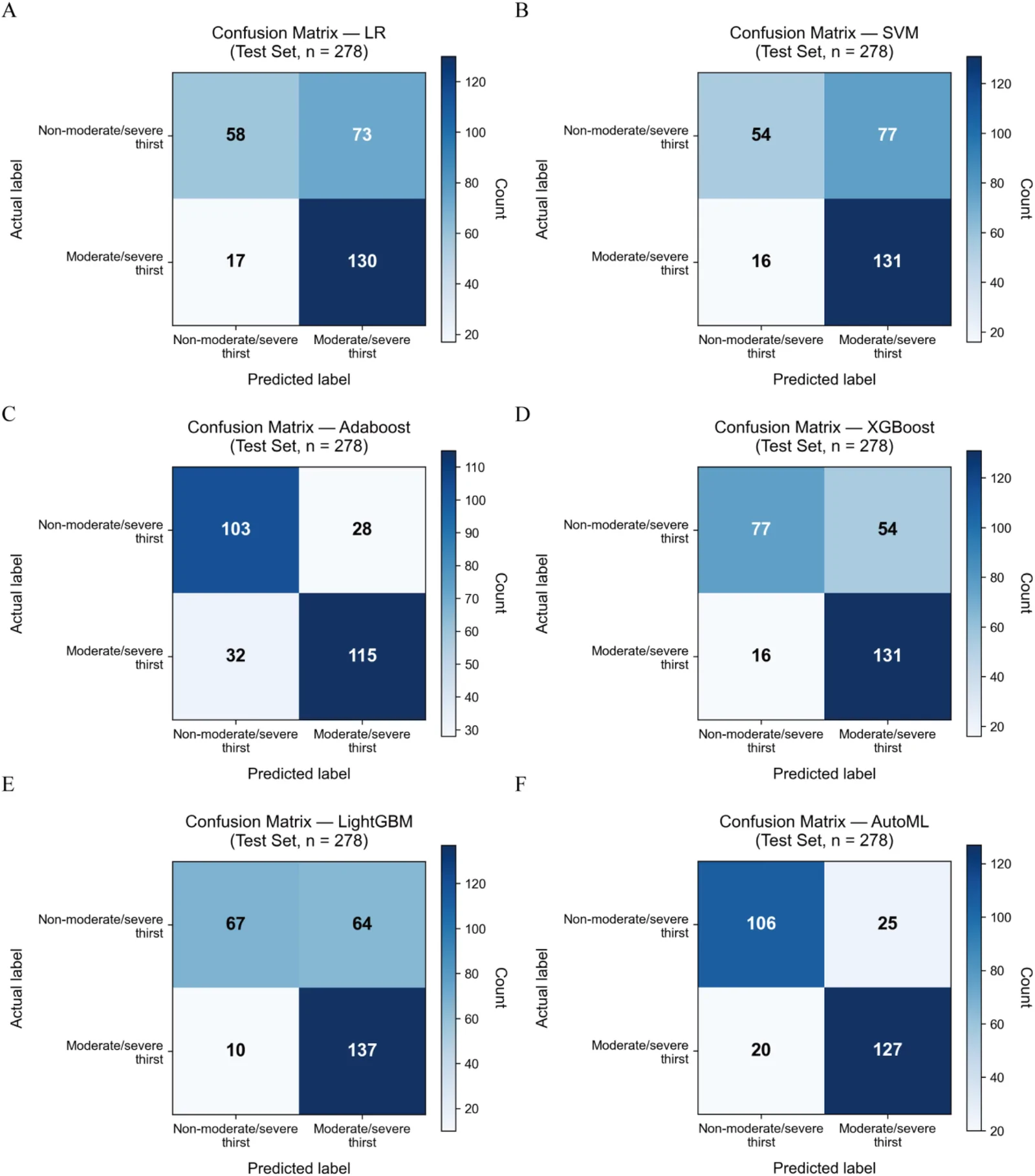
Confusion matrices of prediction results for each model on the test set. **(A)** LR; **(B)** SVM; **(C)** AdaBoost; **(D)** XGBoost; **(E)** LightGBM; **(F)** AutoML.

### 3.4 Temporal validation

We applied the finalized AutoML model, without any modification, to a temporal validation cohort consisting of 152 patients to evaluate its performance stability under temporal shift. The incidence of MSPOT in the temporal validation cohort was 50.7% (77/152), and in the development cohort it was 54.96% (510/928) (*χ*^2^ = 0.973, *P* = 0.324). No statistically significant differences were observed in the outcome and the six predictors of the model between the development cohort and the temporal validation cohort (all *P* > 0.05; see Table 3), suggesting that the case mix remained generally stable over this short interval. The model's discriminative ability remained acceptable (Figures 5A,B): the ROC-AUC was 0.8807 (95% CI: 0.8239–0.9286), and the PR-AUC was 0.8778 (95% CI: 0.8196–0.9283). However, calibration performance showed a clinically important decline and was therefore treated as a central temporal validation finding rather than a minor secondary limitation. The Brier score was 0.1924 (95% CI: 0.1436–0.2434). Calibration assessment of the unmodified model estimated an intercept of −1.2250 (95% CI: −1.8546 to −0.7464) and a slope of 0.5811 (95% CI: 0.4537–0.7897); because both confidence intervals excluded the ideal values of 0 and 1, respectively, these results indicate average risk overestimation and overly extreme predicted probabilities relative to observed outcomes (Figure 5D). At the study-wide prespecified probability threshold of 0.5, the sensitivity was 0.9610 (95% CI: 0.9130–1.0000), the specificity was 0.5733 (95% CI: 0.4603–0.6850), the positive predictive value was 0.6981 (95% CI: 0.6111–0.7851), and the negative predictive value was 0.9348 (95% CI: 0.8542–1.0000) (TP = 74, TN = 43, FP = 32, FN = 3). The reduced specificity and 32 false-positive classifications among 75 patients without MSPOT further suggest that the current probability estimates should not be used directly to trigger probability-based clinical decisions in a new time period. No recalibrated model was fitted for performance claiming in this temporal cohort; any recalibrated version would require evaluation in a subsequent prospectively collected dataset. Decision curve analysis demonstrated that within the threshold probability interval of approximately 0.01–0.74, the model yielded a positive net benefit and outperformed the “treat all” strategy (Figure 5C).

**Table 3:** Baseline characteristics of the development cohort vs. the temporal validation cohort.

Characteristic	Development (*n* = 928)	Temporal (*n* = 152)	Statistic	*P*-value
MSPOT, *n* (%)			*χ*^2^ = 0.973	0.324
Yes	510 (54.96%)	77 (50.66%)	—	—
No	418 (45.04%)	75 (49.34%)	—	—
ERAS, *n* (%)			*χ*^2^ = 0.048	0.827
Yes	424 (45.69%)	68 (44.74%)	—	—
No	504 (54.31%)	84 (55.26%)	—	—
Use of Esmolol, *n* (%)			*χ*^2^ = 0.245	0.621
Yes	312 (33.62%)	48 (31.58%)	—	—
No	616 (66.38%)	104 (68.42%)	—	—
ASA classification, *n* (%)			*χ*^2^ = 0.100	0.992
ASA I	136 (14.66%)	23 (15.13%)	—	—
ASA II	444 (47.84%)	74 (48.68%)	—	—
ASA III	297 (32.00%)	47 (30.92%)	—	—
ASA IV	51 (5.50%)	8 (5.26%)	—	—
Operation time (minutes), M [Q1, Q3]	96.5 [70.2, 122.0]	93.5 [66.0, 119.0]	*Z* = 0.560	0.576
Intraoperative blood loss (mL), M [Q1, Q3]	120.0 [60.0, 200.0]	115.0 [55.0, 195.0]	*Z* = −0.019	0.985
Intraoperative fluid infusion (mL), M [Q1, Q3]	1,550.0 [1,143.2, 1,969.8]	1,520.0 [1,099.8, 1,900.0]	*Z* = 1.424	0.155

**Figure 5:**
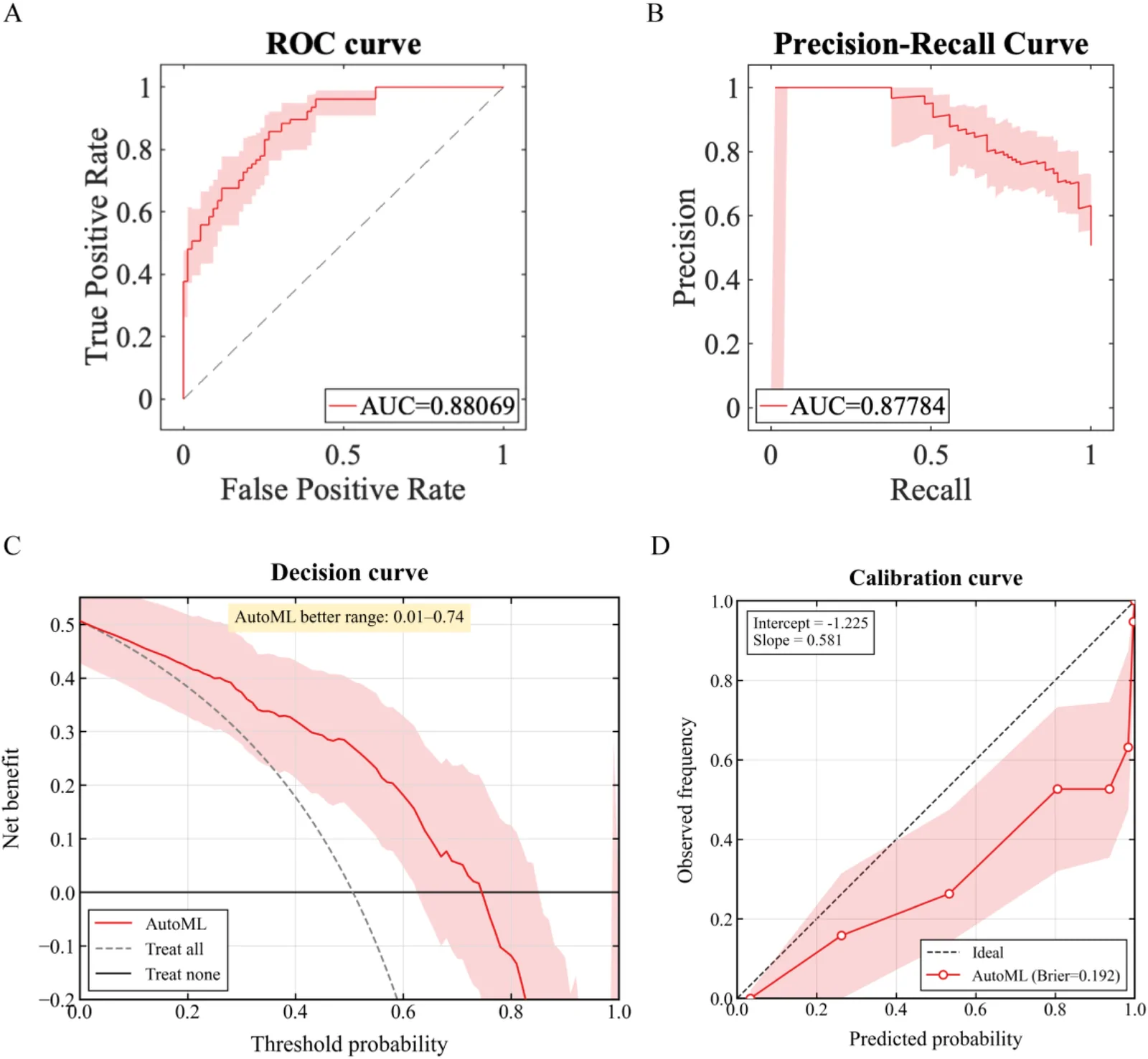
Prediction results for the temporal validation cohort. Confidence intervals were calculated based on 2,000 bootstrap resamples. **(A)** ROC curve; **(B)** PR curve; **(C)** DCA curve; **(D)** Calibration curve. The calibration curve was constructed using decile-based observed event rates.

### 3.5 Analysis of key influencing factors

#### 3.5.1 LASSO regression

As a supplementary consistency analysis conducted separately from the AutoML pipeline, LASSO regression retained 10 variables with non-zero coefficients under the lambda_1SE criterion. All six features selected by AutoML, namely use of esmolol, intraoperative blood loss, operation time, ASA classification, ERAS implementation, and intraoperative fluid infusion volume, also had non-zero coefficients in the LASSO analysis. The complete overlap of these six core features supports the consistency of the AutoML feature-selection result. The four additional variables retained by LASSO were not used to modify, retrain, or expand the final AutoML model, which remained based on the six AutoML-selected features (Figure 6).

**Figure 6:**
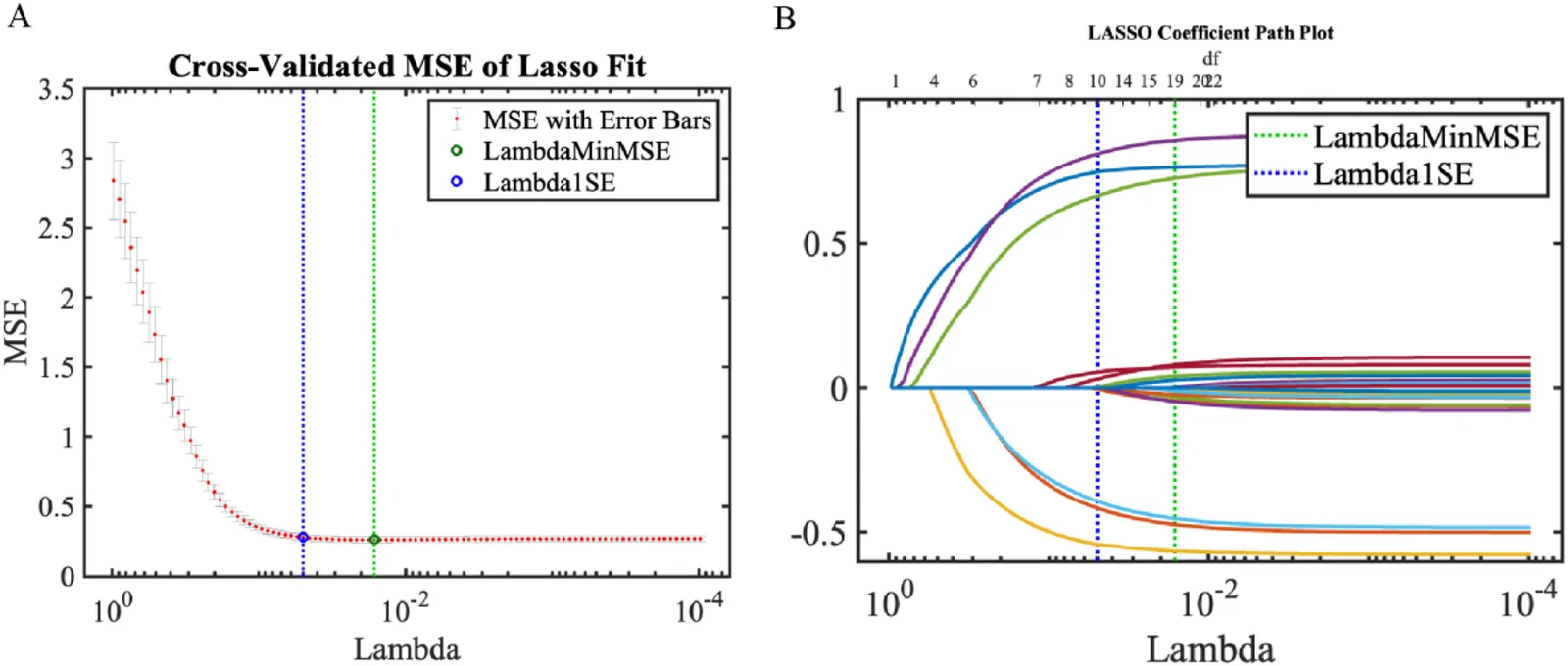
LASSO regression results. **(A)** Cross-validation MSE profile; **(B)** LASSO coefficient trajectories. At lambda_1SE, 10 variables had non-zero coefficients, including all six AutoML-selected features.

#### 3.5.2 SHAP interpretation

SHAP analysis demonstrated the following feature-importance ranking (Figures 7A,B): Use of Esmolol > Intraoperative blood loss > ERAS > Operation time > ASA classification > Intraoperative fluid infusion volume. Cases with higher model-predicted probabilities generally showed right-shifted decision paths, reflecting the cumulative contribution of multiple features to the model output (Figure 7F).

**Figure 7:**
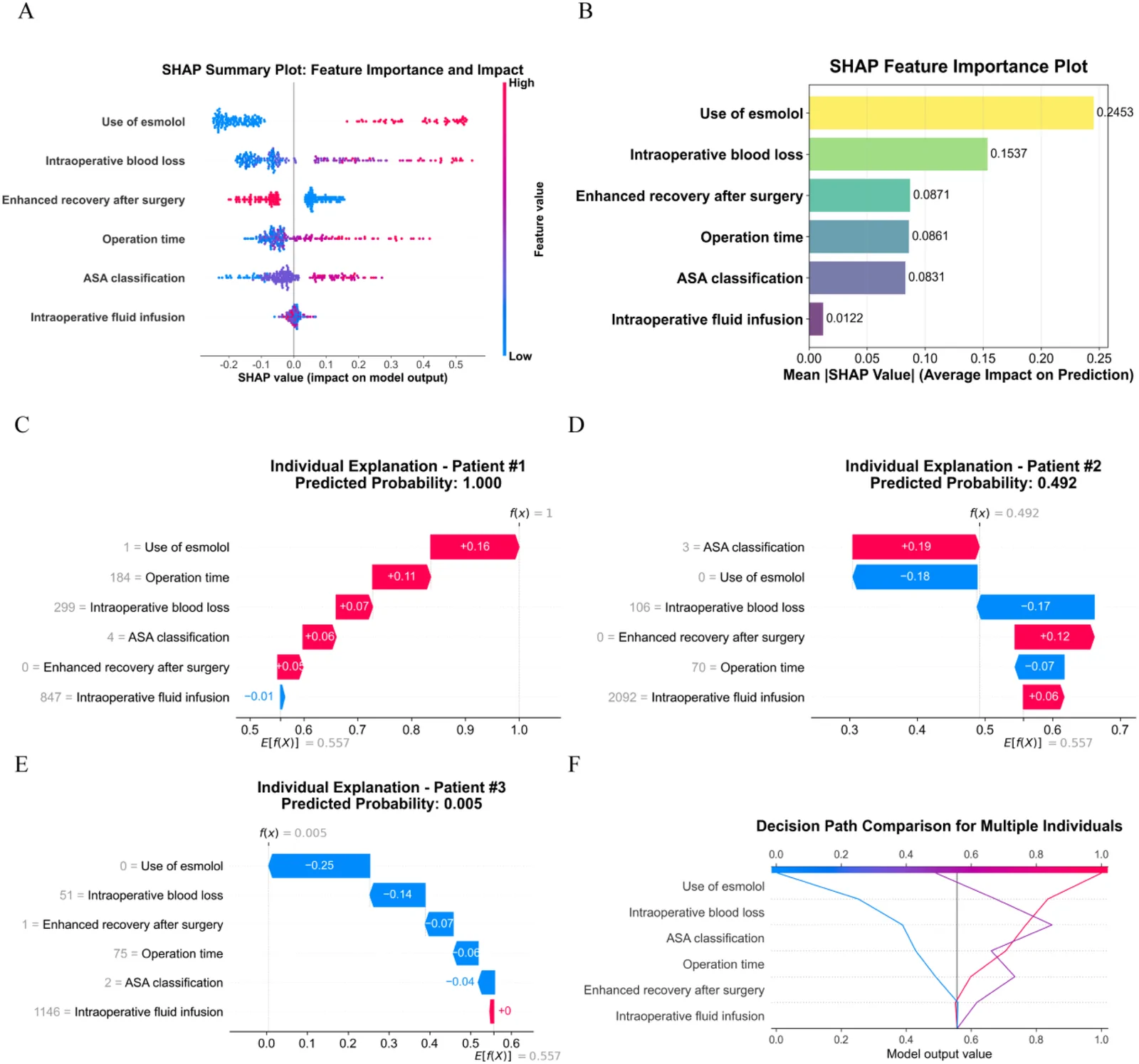
SHAP interpretability analysis. **(A)** The Shapley summary plot; **(B)** The Shapley feature importance plot; **(C)**–**(E)** Waterfall plots illustrate the cumulative contribution process of each feature to individual patient predictions. The baseline value represents the model's average prediction for all patients, while feature contributions show how each feature affects the final prediction (red indicating increased risk, blue indicating decreased risk). The sum of all feature contributions yields the final predicted value; **(F)** The decision path plot compares decision pathways across multiple patients, demonstrating how different feature combinations lead to varying prediction outcomes. The horizontal axis shows predicted probabilities, the vertical axis lists features, and the curved pathways trace decision routes from baseline values to final predictions.

Individual case analyses were performed to illustrate the local interpretability of the model. It should be emphasized that these individual case analyses illustrate how the model allocates its prediction among variables; they do not demonstrate causal relationships between specific clinical factors and postoperative thirst.

Representative case 1, with a predicted probability of 1.000 (ID 1, Figure 7C), involved esmolol administration (use_esmolol = yes), which showed the largest positive SHAP contribution. The patient also had substantial intraoperative blood loss (299 mL, compared with a median of 120 mL) and a relatively long operation time (184 min, compared with a median of 96.5 min). ASA class IV, non-implementation of ERAS, and a relatively low intraoperative fluid infusion volume (847 mL, compared with a median of 1,550 mL) were also present. The combined contribution of these feature values resulted in a model-predicted probability of 1.000.

Representative case 2, with a borderline predicted probability of 0.492 (ID 2, Figure 7D), was slightly below the prespecified classification threshold of 0.5 and was therefore classified as negative. The patient did not receive esmolol, which contributed negatively to the model output in this case. ASA class III produced the largest positive SHAP contribution. The patient had intraoperative blood loss of 106 mL, an operation time of 70 min, no ERAS implementation, and an intraoperative fluid infusion volume of 2,092 mL. The opposing contributions of these features resulted in a prediction close to the classification threshold, illustrating how multiple feature effects jointly influence an individual prediction.

Representative case 3, with a predicted probability of 0.005 (ID 3, Figure 7E), was below the prespecified classification threshold. The patient did not receive esmolol, had intraoperative blood loss of 51 mL, an operation time of 75 min, ASA class II, ERAS implementation, and an intraoperative fluid infusion volume of 1,146 mL. The displayed features contributed predominantly in the negative direction according to the SHAP analysis, and their combined effects resulted in a low model-predicted probability of 0.005.

### 3.6 Clinical decision support system development

As a proof-of-concept to explore potential solutions for barriers in clinical adoption of AI tools requiring programming expertise, we developed a prototype visualization system (Figure 8). Clinicians need only input values for the 6 key prediction features to automatically calculate MSPOT risk and receive clinical recommendations. The recommended workflow is PACU-admission calculation during handover, followed by prioritized direct NRS assessment and early comfort-oriented nursing measures for patients flagged as high risk. Because temporal validation revealed substantial probability miscalibration, the current software should not be interpreted as ready for clinical deployment or probability-threshold-based decision-making; recalibration and prospective validation are required before implementation.

**Figure 8:**
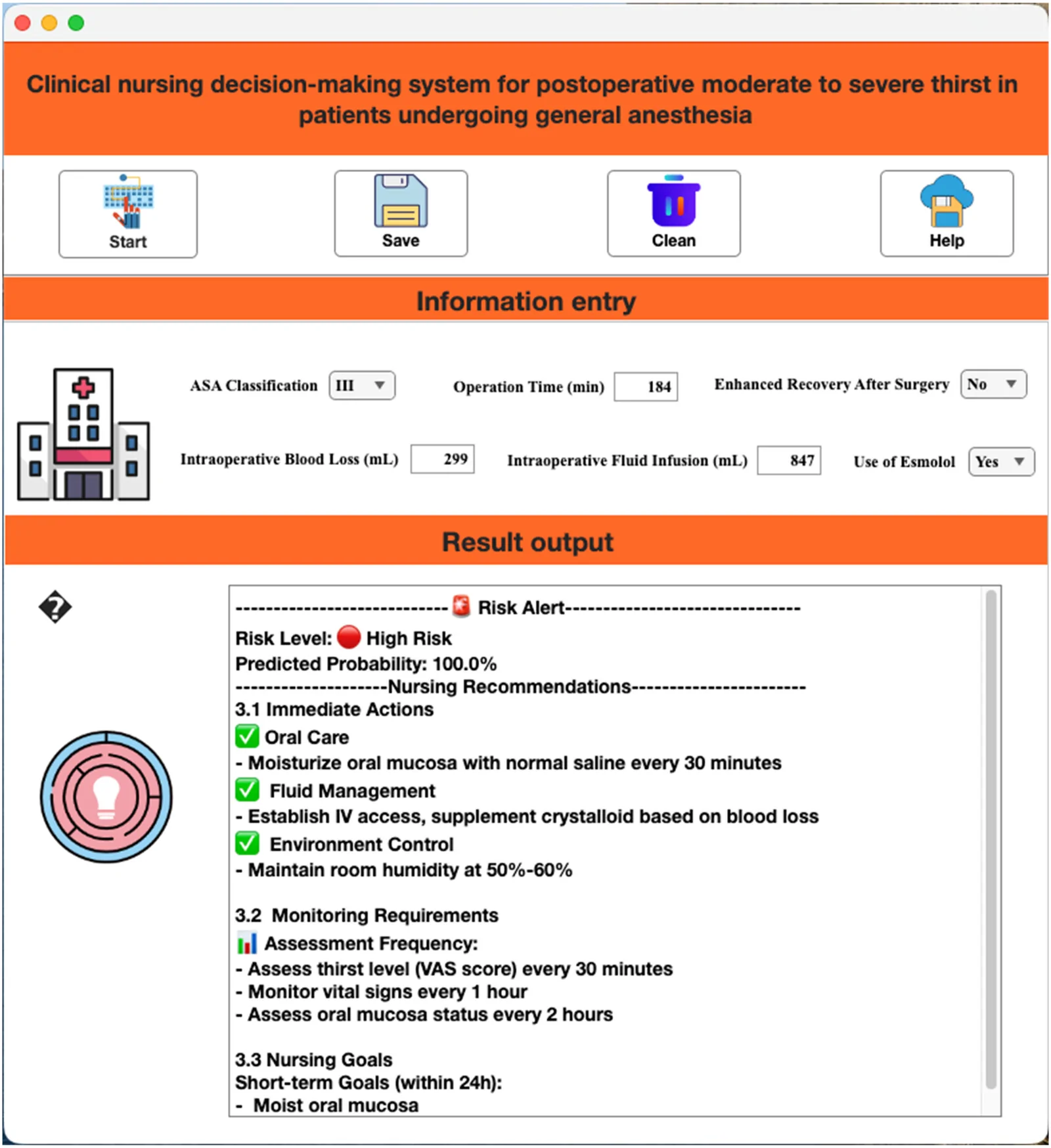
User interface of the software system.

## 4 Discussion

This study successfully established an automated machine learning model based on the Improved Wave Optics Optimizer (IWOO), achieving precise prediction of moderate-to-severe postoperative thirst (MSPOT) in patients undergoing general anesthesia. In the held-out internal test set, the model demonstrated strong discriminative performance (AUC: 0.9053), significantly surpassing the traditional logistic regression model (AUC: 0.7505). This substantial improvement outcome suggests that integrating the automatic feature selection and hyperparameter optimization mechanisms of metaheuristic algorithms can effectively capture the multifactorial nonlinear interaction network driving high-risk MSPOT states, resolving the insufficient predictive accuracy of traditional models caused by preset variables and linear assumptions. The temporal validation results, however, separate discrimination from probability reliability. Although the model maintained acceptable ranking ability in the temporal cohort, the calibration intercept of −1.2250 and slope of 0.5811 showed that predicted probabilities were systematically misaligned with observed risk. Therefore, the present model should be considered useful for exploratory risk stratification and hypothesis generation, but not yet suitable for individualized probability-based decision-making without recalibration. The model is intended for use during the immediate transition from surgery completion to PACU admission. Several final predictors, including total operation time, total blood loss, total fluid infusion, and actual intraoperative esmolol use, become complete only after surgery; accordingly, the score is calculated after anesthesia handover information is available and before or alongside the first standardized NRS thirst assessment. The clinical lead time is short and operational rather than preoperative: the model may help PACU staff identify patients who merit prioritized assessment and comfort-oriented nursing measures during the first minutes of PACU care, particularly when reliable self-report is temporarily delayed. For alert and communicative patients, direct NRS assessment remains the simpler and definitive early screening method; the model is best viewed as a structured adjunct for handover-based triage and workflow prioritization.

Using SHAP interpretability analysis, this study summarized the predictive contribution patterns associated with postoperative moderate-to-severe thirst risk. These SHAP values explain the model's prediction but should be interpreted as associations rather than direct causal effects, as discussed further in the Discussion section. Esmolol use was the strongest predictor in the model. In this cohort, “use of esmolol” referred to continuous intraoperative infusion rather than a single bolus at induction or emergence. Human studies of other beta-adrenoceptor antagonists indicate that beta blockade can alter salivary secretion or composition (20, 21), while salivary function is also influenced by systemic disease and patient-level factors (22). These sources do not establish that intraoperative esmolol directly causes postoperative thirst, and no esmolol-specific salivary measurements were obtained in this study. Esmolol use may also correlate with greater surgical complexity, intraoperative hemodynamic instability, or higher-acuity patient status, creating potential confounding by indication. Accordingly, its SHAP contribution should be interpreted in the context of coexisting risk markers rather than as an isolated pharmacological effect. Regarding the exclusion of clinically plausible xerostomia-associated medications (e.g., atropine, penehyclidine hydrochloride, and ondansetron) from the final six-feature model, the feature-selection process was data-driven and evaluated incremental predictive contribution within training-set cross-validation. Medication-related salivary gland dysfunction varies by drug, dose, exposure duration, concomitant medications, and patient characteristics, and anticholinergic agents are recognized xerogenic exposures (23). Our study did not measure salivary flow or drug-specific pharmacodynamic effects. Therefore, exclusion of these medications from the final model should not be interpreted as evidence that they lack salivary effects; it indicates only that they did not add sufficient incremental predictive value under the present selection procedure. Their lower model contribution may also reflect limited exposure variability or shared information with stronger predictors. Overall, these findings are associative and do not support changes to perioperative medication practice.

The SHAP attribution results also highlighted ASA classification. ASA physical status is a broad clinical classification of preoperative physical fitness and disease burden rather than a thirst-specific physiological measure (24). Its contribution in this model may therefore summarize comorbidity, treatment intensity, and perioperative complexity; it should not be interpreted as evidence that ASA IV status itself causes thirst or establishes reduced fluid-compensation capacity. In Figure 7, the global SHAP plots and waterfall/decision-path visualization summarize predictive patterns without implying causal interaction effects. In contrast, the implementation of Enhanced Recovery After Surgery (ERAS) pathways was associated with lower model-predicted risk, a finding plausibly consistent with ERAS principles of integrated optimization of preoperative fasting management and goal-directed fluid therapy (25, 26). While the observational nature of this study precludes causal inference, these multivariable associations are consistent with a multifactorial prediction involving medication exposures, baseline clinical status, and procedural variables; they do not establish biological mechanisms. Prospective studies with formal interaction testing are needed to determine whether these patterns reflect biological interactions or correlated risk markers. The study recommends focusing risk interventions on controllable factors: for patients identified as high-risk at PACU admission, clinicians should prioritize targeted thirst-relief interventions, such as early oral care, judicious fluid supplementation, and intensified comfort measures, and establish a dynamic monitoring system for blood loss and anesthesia duration in complex surgeries. Because this model incorporates intraoperative variables, its use is limited to postoperative/PACU risk screening. Preoperative targeted prevention would require a separate model developed exclusively from variables available before the intervention. However, no clinical recommendations regarding specific medication use, including esmolol, should be drawn from these observational SHAP analyses, given the inability to exclude confounding by indication in this retrospective design.

Despite methodological innovation and clinical translation important step forward, this study has the following limitations: At the population representativeness level, the single-center design may introduce selection bias—especially the insufficient proportion of ASA level IV patients—limiting the model's applicability in critically ill populations. Because the outcome depended on reliable NRS assessment within 30 min after PACU admission, the present data do not establish model performance in patients with persistent inability to communicate or prolonged deep sedation. At the variable coverage level, the failure to continuously monitor key physiological indicators like serum osmolality and antidiuretic hormone might miss warning signals of occult dehydration states. The data were collected from 2023 to 2024. While recent, clinical practices (e.g., ERAS protocols, anesthetic drug preferences) may evolve, necessitating future model updates and recalibration to maintain its predictive accuracy and relevance. Most importantly, temporal validation demonstrated substantial miscalibration despite acceptable discrimination; therefore, the current individualized probabilities should be interpreted cautiously and should not be used as deployable probability estimates. Recalibration would be necessary before use in a new period or setting, and any recalibrated model would require subsequent evaluation in another prospectively collected dataset rather than being validated in the same temporal cohort used for recalibration. At the technical implementation level, To demonstrate proof-of-concept, we developed a prototype clinical decision support tool using MATLAB App Designer. Future work will transform this into a lightweight web application for broader deployment. The current software remains a research prototype and requires prospective validation before being considered for clinical application. Future research should focus on three directions: (1) Model validation: Conduct a multicenter prospective cohort study to validate the model's generalizability in vulnerable populations (e.g., elderly patients, hepatic/renal dysfunction). (2) Mechanism exploration: Integrate salivary gland ultrasound radiomics and metabolomics technologies to build an “imaging–biomarker–machine learning” multidimensional early-warning system. (3) Clinical intervention: Design and test PACU-based model-assisted workflows, such as prioritized NRS reassessment, oral moisturizing, safety-appropriate thirst-relief bundles, and nursing resource allocation for patients flagged as high risk at PACU admission. The temporal validation cohort provides supportive evidence indicating that the model's discriminative ability was largely preserved under temporal drift within the same hospital over a short interval. However, this cohort was single-center in nature, had a limited sample size, and was temporally adjacent to the model development period; therefore, it cannot demonstrate geographical transportability or multi-center generalizability. The observed calibration failure also means that the software interface should avoid presenting the current values as clinically actionable individualized risks until recalibration and prospective evaluation have been completed. Meanwhile, although the current internal validation combines cross-validation with a held-out internal test set, it does not employ fully nested cross-validation or bootstrapping to quantify performance variance. In subsequent multicenter validation, we will adopt five-fold nested cross-validation to provide higher-level stability evidence. Furthermore, regarding missing data handling, although the proportion of missing values for all predictors was below 5% and the distributions remained stable after imputation, single imputation methods inherently cannot reflect the uncertainty of missing values, which may result in a slight underestimation of standard errors. In future multicenter prospective studies, we will employ multiple imputation combined with sensitivity analysis to more rigorously assess the potential impact of missing-data mechanisms, thereby further strengthening the evidence.

## 5 Conclusion

In summary, this study addressed accuracy bottlenecks and explored potential approaches to clinical translation challenges in postoperative thirst risk prediction by innovatively integrating an improved AutoML algorithm with explainable AI technology, providing a theoretical basis for individualized thirst risk management during PACU admission. The model showed strong discrimination in the held-out internal test set and acceptable discrimination in same-center temporal validation, but temporal miscalibration limits the reliability of its individualized probability estimates. The developed clinical decision system demonstrates a proof-of-concept prototype that might, after rigorous prospective validation, help translate scientific findings into actionable tools, with intended use as a PACU handover-based risk-screening aid. Before clinical deployment, the model will require recalibration and subsequent evaluation in a separate prospectively collected dataset. Future efforts will continuously optimize the model's universality and real-time monitoring capabilities, promoting the transformation of perioperative thirst management into a precise, responsive PACU care model.

## Data Availability

The datasets presented in this article are not readily available because data cannot be shared publicly because of patient privacy. Data are available from the First Affiliated Hospital with Nanjing Medical University Institutional Data Access (contact via whrjsph@njmu.edu.cn) for researchers who meet the criteria for access to confidential data. Requests to access the datasets should be directed to Haoran Wang, whrjsph@njmu.edu.cn. The analytical code used for data preprocessing, feature selection, model training, validation, calibration assessment, and figure generation is available from the corresponding author upon reasonable request, subject to institutional data-governance and patient-privacy requirements. No formal prospective study protocol was prepared or registered before study initiation.
