# Supplementary material for: Prediction of moderate-to-severe postoperative thirst in general anesthesia patients based on automated machine learning and clinical nursing translation
Source: Front Med (Lausanne). 2026 Sep 14;13:1846230. doi: 10.3389/fmed.2026.1846230 (PMC13617033; doi:10.3389/fmed.2026.1846230)
Supplement: Supplementary file 1 [file Datasheet1.docx]

**Appendix A. AutoML Pseudo-Code and Reproducibility Details**

function [best_features, best_params] = IWOO_AutoML(X_train, y_train, max_iter, pop_size)

% Joint encoding: binary feature mask + continuous XGBoost hyperparams

n_features = size(X_train, 2);

n_params = 6; % e.g., n_estimators, max_depth, learning_rate, subsample, colsample_bytree, gamma

dim = n_features + n_params;

% Initialize population via Logistic chaotic map (innovation 1)

population = logistic_chaotic_init(pop_size, dim);

% Evaluate fitness: maximize 5-fold CV AUC

fitness = arrayfun(@(i) cv_auc(population(i,:), X_train, y_train), 1:pop_size);

[best_fit, best_idx] = max(fitness);

best_sol = population(best_idx, :);

for iter = 1:max_iter

% Diffraction phase: global exploration (original WOO operator)

pop_diff = diffraction_update(population);

% Interference phase: local exploitation enhanced by Lévy flight (innovation 2)

pop_int = interference_levy_update(pop_diff);

% Ensure binary features are 0/1, hyperparameters within bounds

pop_new = boundary_repair(pop_int);

% Reevaluate fitness

new_fitness = arrayfun(@(i) cv_auc(pop_new(i,:), X_train, y_train), 1:pop_size);

% Greedy update

[population, fitness] = greedy_select(population, fitness, pop_new, new_fitness);

[iter_best, idx] = max(fitness);

if iter_best > best_fit

best_fit = iter_best;

best_sol = population(idx, :);

end

% Stall check and early termination

if stall_generations_exceed(threshold)

break;

end

end

best_features = logical(round(best_sol(1:n_features))); % selected features

best_params = best_sol(n_features+1 : end); % optimal hyperparameters

end

**The following tables specify the data-isolation rules, optimization settings, comparison-model grids, encoded variables, medication screening procedure, and model-artifact requirements used to support reproducibility. All preprocessing parameters were estimated from the relevant training data and then applied unchanged to held-out data.**

**Leakage-control sequence**

1. Split the development cohort into training and independent test sets before estimating any imputation or encoding parameters.

2. Within each inner-CV fold, fit the imputation and encoding maps on the inner-training portion only.

3. Apply the fitted maps unchanged to the inner-validation portion, the independent test set, and the temporal validation cohort.

4. Perform medication screening and all outcome-based feature selection only within the training resampling process; the prespecified 79-to-12 medication screen used no MSPOT outcome information.

**Table S3. Data Preprocessing and Reproducibility Settings**

| **Component** | **Specification** |
| --- | --- |
| Dataset partition | Stratified 70:30 split; training set n=650, independent test set n=278, and temporal validation cohort n=152 |
| Preprocessing fit scope | Imputation, encoding, scaling , feature selection, and tuning were fitted on training data only; inner-CV transformations were refit in each training fold |
| Continuous-variable imputation | Training-fold median; the value was applied unchanged to the corresponding validation, test, and temporal records |
| Categorical-variable imputation | Training-fold mode; the value was applied unchanged to the corresponding validation, test, and temporal records |
| One-hot encoding | Category levels were learned from the training fold; the resulting column order was fixed for all held-out cohorts |
| Feature-selection scope | Training set only; neither test-set nor temporal-cohort outcomes entered feature selection or hyperparameter tuning |
| Fitness objective | Maximize the mean ROC-AUC across stratified 5-fold cross-validation |
| IWOO population size | 30 |
| Maximum iterations | 200 |
| Early stopping | Stop after 15 consecutive generations with an improvement smaller than 1e-6 |
| Data-split random seed | 20240117 |
| Cross-validation random seed | 20240118 |
| IWOO random seed | 20240119 |
| Bootstrap random seed | 20240120; 2,000 resamples for 95% confidence intervals |
| Master seed in the feature-selection comparison script | 2023; MATLAB rng |
| Software | MATLAB R2024b; Statistics and Machine Learning Toolbox 24.2; Optimization Toolbox 24.2; IBM SPSS Statistics 26.0 |

**Table S4. Final AutoML (IWOO-XGBoost) Configuration**

| **Parameter** | **Search range** | **Final value** |
| --- | --- | --- |
| Base learner | XGBoost binary classifier | XGBoost binary classifier |
| n_estimators | 50-500 | 286 |
| max_depth | 3-15 | 4 |
| learning_rate | 0.01-0.30 | 0.047 |
| subsample | 0.60-1.00 | 0.82 |
| colsample_bytree | 0.60-1.00 | 0.76 |
| gamma | 0-0.50 | 0.09 |
| Selected feature blocks | 28 candidate variables | 6 |
| Optimization objective | Mean 5-fold ROC-AUC | Maximize |

**Table S5. Prespecified Hyperparameter Grids for Comparison Models**

| **Model** | **Hyperparameter** | **Candidate values** |
| --- | --- | --- |
| LR | Learner | logistic |
| LR | Regularization | ridge; lasso |
| LR | Lambda | 1e-4; 1e-3; 1e-2; 1e-1; 1; 10 |
| SVM | KernelFunction | linear; rbf |
| SVM | BoxConstraint | 0.01; 0.1; 1; 10; 100 |
| SVM | KernelScale | auto; 0.1; 1; 10 |
| AdaBoost | NumLearningCycles | 50; 100; 200; 300 |
| AdaBoost | LearnRate | 0.01; 0.05; 0.10; 0.50; 1.00 |
| AdaBoost | MaxNumSplits | 1; 3; 5; 10 |
| XGBoost | n_estimators | 100; 200; 300; 500 |
| XGBoost | max_depth | 3; 4; 6; 8 |
| XGBoost | learning_rate | 0.01; 0.05; 0.10; 0.20 |
| XGBoost | subsample | 0.70; 0.85; 1.00 |
| XGBoost | colsample_bytree | 0.70; 0.85; 1.00 |
| XGBoost | gamma | 0; 0.10; 0.30 |
| LightGBM | NumLearningCycles | 100; 200; 300; 500 |
| LightGBM | MaxNumSplits | 3; 5; 7; 10 |
| LightGBM | LearnRate | 0.01; 0.05; 0.10; 0.20 |
| LightGBM | MinLeafSize | 5; 10; 20 |
| LightGBM | BagFraction | 0.70; 0.85; 1.00 |

**Table S6. One-Hot Encoding Map and Final Feature Blocks**

| **Original variable** | **Observed levels** | **Model columns** | **Selection status** |
| --- | --- | --- | --- |
| Sex | Female; Male | Sex_Male (Female reference) | Not selected |
| Education level | Primary school or below; Middle school; High school; College or above | Education_Middle_school; Education_High_school; Education_College_or_above (Primary school or below reference) | Not selected |
| ERAS implementation | No; Yes | ERAS_Yes (No reference) | Selected |
| Surgical approach | Open; Laparoscopic; Endoscopic; Other | Surgery_Laparoscopic; Surgery_Endoscopic; Surgery_Other (Open reference) | Not selected |
| Intraoperative fluid type | Crystalloid only; Colloid only; Both | Fluid_Colloid_only; Fluid_Both (Crystalloid only reference) | Not selected |
| ASA classification | ASA I; ASA II; ASA III; ASA IV | ASA_II; ASA_III; ASA_IV (ASA I reference) | Selected |
| Intubation method | Endotracheal intubation; Laryngeal mask; Other | Intubation_Laryngeal_mask; Intubation_Other (Endotracheal intubation reference) | Not selected |
| Medication indicators | 0/1 indicator for each of 12 medications | Original binary medication columns; no additional dummy columns | Use_of_Esmolol selected; other 11 not selected |
| Continuous variables | Age; water intake; fasting time; operation time; anesthesia duration; fluid volume; blood loss; urine output | Original numeric columns after training-fold imputation | Operation time; intraoperative fluid volume; intraoperative blood loss selected |

**Table S7. Prespecified Screening of 79 Perioperative Medications**

| **Screening stage** | **Operational rule** | **Result** |
| --- | --- | --- |
| Initial medication pool | All 79 medication indicators recorded in the perioperative electronic medical record | 79 candidate medications |
| Clinical plausibility | Prespecified pharmacologic relevance to salivary secretion, oral dryness, fluid balance, or routine perioperative exposure | Retain clinically plausible agents |
| Data traceability | Medication identity, administration record, and binary exposure status were available; duplicate salt names were harmonized | Retain traceable variables |
| Exposure variation | Nonzero exposure and sufficient variation were assessed descriptively without using MSPOT labels | Retain variables with usable exposure variation |
| Outcome independence | No MSPOT outcome, univariable P value, outcome association estimate, or post-outcome information was used for this screen | Screen completed before model fitting |
| Final candidate list | Propofol; atropine; nalbuphine hydrochloride; hydrocodone hydrochloride; penehyclidine hydrochloride; butorphanol tartrate; esmolol; calcium gluconate; isoprenaline; furosemide; dexmedetomidine hydrochloride; ondansetron hydrochloride | 12 medication variables |
