# Supplementary material for: Prediction of moderate-to-severe postoperative thirst in general anesthesia patients based on automated machine learning and clinical nursing translation
Source: Front Med (Lausanne). 2026 Sep 14;13:1846230. doi: 10.3389/fmed.2026.1846230 (PMC13617033; doi:10.3389/fmed.2026.1846230)
Supplement: Supplementary file 2 [file Datasheet2.docx]

**Appendix B. CEC2022 Benchmark Validation of IWOO**

**This appendix reports the CEC2022 benchmark validation of IWOO. The complete numerical results are provided in Table S1, and the reproducibility settings are summarized in Table S2.**

To validate the optimization capability of the improved IWOO algorithm, we compared it with the original WOO, WOA, GWO, PSO, GA, GA-PSO, and GA-ACO algorithms. All 12 benchmark functions in the CEC2022 test suite were used. The dimensionality of each function was set to 10, the population size was 30, and the maximum number of iterations was 500. Each algorithm was independently run 30 times to ensure statistical reliability. Boxplots were generated to assess optimization stability across the 30 runs. IWOO performed favorably on most benchmark functions and showed greater stability than the original WOO and the other comparison algorithms (Figure S1A). Convergence curves further indicated that IWOO converged faster and was less likely to become trapped in local optima during the iterations (Figure S1B). These results support the superior global-search performance and convergence efficiency of IWOO. Complete results are presented in Table S1.

**Figure S1. Benchmarking of the Improved Wave Optics Optimizer**

Note: A, boxplots of the optimization results after 30 independent runs of each algorithm on the CEC2022 test functions, illustrating optimization stability and robustness; B, convergence curves during optimization, illustrating convergence speed and the ability to avoid local optima.

**Table S1. CEC2022 Benchmark Results (Dimension = 10)**

| **Function** | **Algorithm** | **Best** | **Standard Deviation** | **Mean** | **Median** | **Worst** |
| --- | --- | --- | --- | --- | --- | --- |
| F1 | IWOO | 300.0138 | 0.1297 | 300.1427 | 300.1223 | 300.5028 |
|  | WOO | 312.8534 | 335.6603 | 636.3218 | 524.5486 | 1428.796 |
|  | WOA | 4922.3228 | 10865.1829 | 23253.7608 | 22309.2244 | 41071.1739 |
|  | GWO | 391.4545 | 1866.7853 | 1894.9866 | 566.1254 | 5598.4183 |
|  | PSO | 6281.1532 | 14180.9937 | 25972.5239 | 22352.5719 | 52406.8265 |
|  | GA | 391.4837 | 1155.9208 | 1525.5795 | 1592.615 | 4305.2577 |
|  | GA-PSO | 309.3971 | 27.1552 | 335.5115 | 332.0569 | 425.1487 |
|  | GA-ACO | 343.6674 | 159.2868 | 540.7067 | 487.709 | 814.255 |
| F2 | IWOO | 400.0012 | 3.5084 | 405.4341 | 405.8253 | 408.9161 |
|  | WOO | 400.6318 | 85.4987 | 461.2777 | 431.0153 | 693.9782 |
|  | WOA | 400.8556 | 35.8756 | 439.2301 | 426.2816 | 500.47 |
|  | GWO | 400.5584 | 28.7838 | 429.4939 | 411.2769 | 490.0328 |
|  | PSO | 402.5638 | 37.1414 | 433.2447 | 412.176 | 514.6038 |
|  | GA | 400.2305 | 28.4257 | 434.5364 | 426.9582 | 474.2928 |
|  | GA-PSO | 400.2815 | 29.5423 | 422.8666 | 409.1366 | 488.4267 |
|  | GA-ACO | 407.6273 | 30.3951 | 445.2275 | 457.0781 | 477.0138 |
| F3 | IWOO | 600.2338 | 0.67693 | 600.9846 | 600.8553 | 602.791 |
|  | WOO | 616.266 | 15.4123 | 639.0157 | 638.7731 | 667.1081 |
|  | WOA | 618.0649 | 17.1818 | 638.5139 | 637.2167 | 690.9065 |
|  | GWO | 600.1157 | 1.1812 | 601.2705 | 601.0263 | 604.581 |
|  | PSO | 607.5248 | 12.9393 | 639.2754 | 638.8041 | 666.4319 |
|  | GA | 602.4681 | 8.2887 | 615.1983 | 611.6406 | 632.5426 |
|  | GA-PSO | 600.7172 | 7.3608 | 607.929 | 606.3479 | 630.9335 |
|  | GA-ACO | 603.0783 | 6.4883 | 609.3604 | 607.5029 | 625.651 |
| F4 | IWOO | 816.1022 | 9.674 | 833.1388 | 832.015 | 847.7287 |
|  | WOO | 805.0919 | 9.7433 | 828.7655 | 828.0967 | 850.0337 |
|  | WOA | 824.1252 | 15.2864 | 842.4477 | 840.016 | 876.1653 |
|  | GWO | 805.6713 | 7.3115 | 814.0967 | 812.4137 | 834.9465 |
|  | PSO | 816.5909 | 15.7052 | 841.8426 | 839.9494 | 880.9707 |
|  | GA | 811.1832 | 10.6679 | 825.998 | 824.6825 | 844.6838 |
|  | GA-PSO | 814.4579 | 7.693 | 823.8787 | 822.1819 | 839.3705 |
|  | GA-ACO | 812.9737 | 5.0791 | 821.5148 | 822.1066 | 833.4375 |
| F5 | IWOO | 900.1799 | 2.736 | 902.623 | 901.8212 | 909.9386 |
|  | WOO | 1183.1183 | 144.7529 | 1460.6591 | 1482.0469 | 1789.5884 |
|  | WOA | 993.5173 | 294.1918 | 1424.8012 | 1347.7688 | 2206.4751 |
|  | GWO | 900.123 | 9.9803 | 905.5806 | 901.2638 | 937.5364 |
|  | PSO | 1081.8655 | 438.026 | 1540.0657 | 1408.3315 | 2990.3333 |
|  | GA | 902.2011 | 167.7827 | 1058.8618 | 998.2103 | 1441.4105 |
|  | GA-PSO | 900.4725 | 158.8394 | 984.6692 | 914.5475 | 1477.2956 |
|  | GA-ACO | 904.0853 | 20.5761 | 925.3703 | 922.9462 | 970.6578 |
| F6 | IWOO | 1808.8795 | 42.0395 | 1845.28 | 1832.9231 | 1965.349 |
|  | WOO | 1883.6904 | 1655.652 | 3897.9468 | 3517.2336 | 7249.4065 |
|  | WOA | 2340.6904 | 1838.5271 | 4855.0685 | 4934.0315 | 8169.377 |
|  | GWO | 2042.1876 | 2322.923 | 6235.4001 | 5832.2947 | 9339.3682 |
|  | PSO | 2211.449 | 1827.4792 | 4626.6433 | 4115.4911 | 8059.5676 |
|  | GA | 1996.198 | 1799.2051 | 4713.7782 | 4761.8363 | 8214.3331 |
|  | GA-PSO | 3017.0773 | 6133.9342 | 13354.3453 | 13203.5546 | 23832.4827 |
|  | GA-ACO | 3373.2702 | 11527.4838 | 21884.1805 | 20086.2444 | 44205.8409 |
| F7 | IWOO | 2022.1918 | 3.0365 | 2025.7172 | 2024.8734 | 2035.8398 |
|  | WOO | 2045.7756 | 15.7891 | 2067.4577 | 2064.7262 | 2097.5706 |
|  | WOA | 2034.1771 | 28.7216 | 2076.7217 | 2069.202 | 2142.5643 |
|  | GWO | 2010.0542 | 9.4839 | 2025.321 | 2024.3326 | 2045.3884 |
|  | PSO | 2036.7053 | 36.4646 | 2084.309 | 2075.1489 | 2160.9414 |
|  | GA | 2021.4648 | 20.569 | 2047.2898 | 2042.2262 | 2104.4481 |
|  | GA-PSO | 2021.607 | 8.3032 | 2032.7247 | 2031.3547 | 2047.6492 |
|  | GA-ACO | 2027.38 | 6.529 | 2038.9128 | 2038.4031 | 2052.3378 |
| F8 | IWOO | 2215.3043 | 3.7982 | 2224.3305 | 2224.974 | 2229.6842 |
|  | WOO | 2213.3072 | 8.7467 | 2229.7958 | 2228.2771 | 2259.3605 |
|  | WOA | 2223.8256 | 14.4031 | 2240.2789 | 2236.2675 | 2274.5955 |
|  | GWO | 2221.9647 | 3.3504 | 2226.0439 | 2225.1476 | 2231.3369 |
|  | PSO | 2226.0354 | 6.2821 | 2234.2641 | 2232.1597 | 2244.6926 |
|  | GA | 2210.0198 | 5.0085 | 2225.9604 | 2226.6776 | 2234.5419 |
|  | GA-PSO | 2210.6575 | 4.5633 | 2226.2978 | 2226.7579 | 2232.3433 |
|  | GA-ACO | 2216.339 | 3.6469 | 2227.2047 | 2227.0837 | 2232.2312 |
| F9 | IWOO | 2529.2844 | 1.09E-09 | 2529.2844 | 2529.2844 | 2529.2844 |
|  | WOO | 2557.7239 | 37.0443 | 2612.9158 | 2600.214 | 2677.3819 |
|  | WOA | 2540.2281 | 50.3036 | 2612.2343 | 2604.5629 | 2697.0157 |
|  | GWO | 2529.6568 | 32.7559 | 2575.4592 | 2568.8045 | 2626.9338 |
|  | PSO | 2533.4744 | 50.0288 | 2613.4877 | 2612.8458 | 2704.9762 |
|  | GA | 2529.2972 | 30.6591 | 2570.1564 | 2570.4775 | 2629.5436 |
|  | GA-PSO | 2529.7596 | 34.521 | 2554.1993 | 2546.0188 | 2676.3075 |
|  | GA-ACO | 2529.519 | 9.7519 | 2537.0476 | 2534.1825 | 2563.3951 |
| F10 | IWOO | 2500.3115 | 0.09508 | 2500.4782 | 2500.4783 | 2500.6485 |
|  | WOO | 2500.7387 | 64.5043 | 2550.9624 | 2501.536 | 2645.9421 |
|  | WOA | 2500.5762 | 91.8753 | 2583.9277 | 2562.4694 | 2780.2553 |
|  | GWO | 2500.3378 | 69.331 | 2556.5282 | 2500.5853 | 2724.0147 |
|  | PSO | 2500.5933 | 282.3251 | 2663.8044 | 2501.5349 | 3442.5977 |
|  | GA | 2500.3584 | 67.3272 | 2573.1013 | 2616.288 | 2656.8409 |
|  | GA-PSO | 2500.4213 | 56.1759 | 2534.4121 | 2500.6403 | 2633.9439 |
|  | GA-ACO | 2500.4692 | 5.7436 | 2502.0137 | 2500.6337 | 2525.0228 |
| F11 | IWOO | 2602.7105 | 5.4901 | 2607.6881 | 2605.6447 | 2621.9561 |
|  | WOO | 2607.8919 | 87.3918 | 2740.4066 | 2750.6633 | 2925.0512 |
|  | WOA | 2729.2719 | 96.2702 | 2996.8447 | 2985.1854 | 3216.5787 |
|  | GWO | 2748.3429 | 123.4524 | 2970.6872 | 2937.409 | 3213.2893 |
|  | PSO | 2714.9712 | 93.7689 | 2966.2163 | 2985.0683 | 3055.2871 |
|  | GA | 2604.3789 | 209.3532 | 2874.8662 | 2773.3807 | 3307.7216 |
|  | GA-PSO | 2628.5823 | 186.3729 | 2704.976 | 2642.9471 | 3217.1286 |
|  | GA-ACO | 2647.9012 | 23.246 | 2675.6835 | 2668.1713 | 2738.6063 |
| F12 | IWOO | 2858.6202 | 1.4594 | 2860.487 | 2861.4049 | 2863.6055 |
|  | WOO | 2865.8295 | 53.7247 | 2925.2637 | 2902.7627 | 3041.3977 |
|  | WOA | 2862.5806 | 54.3216 | 2894.8869 | 2873.2994 | 3089.7018 |
|  | GWO | 2862.7647 | 4.8816 | 2866.9329 | 2865.149 | 2881.6016 |
|  | PSO | 2865.3155 | 69.6964 | 2918.2026 | 2893.261 | 3138.9377 |
|  | GA | 2861.4205 | 12.2471 | 2871.1381 | 2865.5922 | 2906.5292 |
|  | GA-PSO | 2862.6597 | 8.7539 | 2869.0962 | 2865.6847 | 2895.7918 |
|  | GA-ACO | 2863.1959 | 7.9231 | 2867.698 | 2864.736 | 2891.0546 |

**Table S2. CEC2022 Benchmark Protocol and Algorithm Settings**

| **Item** | **Specification** |
| --- | --- |
| Benchmark suite | CEC2022 test functions F1-F12 |
| Optimization direction | Minimization of the benchmark objective function |
| Search dimension | 10 |
| Population size | 30 |
| Maximum iterations | 500 |
| Independent runs | 30 runs for each function and algorithm |
| Compared algorithms | IWOO, WOO, WOA, GWO, PSO, GA, GA-PSO, and GA-ACO |
| IWOO initialization | Logistic chaotic mapping |
| IWOO local search | Interference update enhanced with a Levy-flight perturbation |
| Reported statistics | Best, standard deviation, mean, median, and worst objective values |
| Randomization | Fixed MATLAB random-number stream; master seed 2023 |
| Graphical outputs | Optimization-result boxplots (Figure S1A) and convergence curves (Figure S1B) |
